# In Vitro Immune Response of Mononuclear Cells to Multidrug-Resistant *Escherichia coli*

**DOI:** 10.3390/microorganisms13051164

**Published:** 2025-05-20

**Authors:** Berta Cuyàs, Elisabet Cantó, Elisabet Sanchez-Ardid, Elisenda Miró, Edilmar Alvarado-Tapias, Eva Román, Maria Poca, Ferran Navarro, Andreu Ferrero-Gregori, Maria Àngels Escorsell, Silvia Vidal, German Soriano

**Affiliations:** 1 Department of Gastroenterology, Hospital de la Santa Creu i Sant Pau, C/Mas Casanovas 90, 08041 Barcelona, Spain; bcuyas@santpau.cat (B.C.);; 2 Department of Medicine, Universitat Autònoma de Barcelona, 08023 Barcelona, Spain; 3 Centro de Investigación Biomédica en Red de Enfermedades Hepáticas y Digestivas (CIBERehd), Instituto de Salud Carlos III, 28029 Madrid, Spain; 4 Inflammatory Diseases Department, Institut de Recerca Sant Pau (IR Sant Pau), 08041 Barcelona, Spain; 5 Department of Microbiology, Hospital de la Santa Creu i Sant Pau, C/Mas Casanovas 90, 08041 Barcelona, Spain; 6 Department of Genetics and Microbiology, Institut de Recerca Sant Pau (IR Sant Pau), Universitat Autònoma de Barcelona, 08041 Barcelona, Spain

**Keywords:** resistance, cirrhosis, immune response, MDRO, *Escherichia coli*, cytokines

## Abstract

Infections caused by multidrug-resistant organisms (MDRO) are linked to poor outcomes, particularly in patients with cirrhosis. The underlying mechanisms are not fully understood and may involve a different immune response against MDRO. This study aimed to compare the in vitro immune response between multidrug-resistant (MDR) *Escherichia coli* and antibiotic-susceptible *E. coli* strains. Surface protein extract and DNA extract were obtained from MDR *E. coli* (n = 6) and antibiotic-susceptible *E. coli* (n = 6) strains isolated from infected patients with cirrhosis. The extracts were used to stimulate in vitro peripheral blood mononuclear cells from healthy donors. After 48 h, cytokine levels (IFN-γ, IL-1β, IL-10, IL-12p70, MCP-1, IL-8, IL-6, MIP-1α, and MIP-1β) were measured. We observed no significant differences in cytokine production between MDR and susceptible strains. However, we identified notable interindividual variability in cytokine production for most of the cytokines studied. Only IFN-γ and IL-6 in surface extract and MCP-1 in DNA extract showed similar levels across all donors. We conclude that the cytokine profiles induced by MDR *E. coli* in vitro were similar to those in susceptible strains. These findings suggest that the poor prognosis associated with MDR *E. coli* infections is not due to a differential immune response but rather to other factors.

## 1. Introduction

Antimicrobial resistance is one of the most critical threats to global public health. It is directly responsible for over one million deaths annually worldwide, with the total number expected to exceed 29 million over the next 25 years [1]. This problem is particularly relevant in vulnerable populations, such as patients with cirrhosis. These patients present a spectrum of immune alterations known as cirrhosis-associated immune dysfunction (CAID). CAID is characterized by immune deficiencies and a proinflammatory state. The immune deficiencies, together with microbiome dysbiosis, altered intestinal permeability, and gut bacterial translocation leads to an increase in the incidence of infections [2,3]. As a consequence, infections are a major cause of morbidity and mortality in patients with cirrhosis [4]. In recent years, the prevalence of multidrug-resistant organisms (MDRO) has increased notably in patients with cirrhosis as the result of frequent antibiotic exposure, recurrent hospitalizations, and invasive procedures [5]. Infections caused by MDRO now occur in approximately one-third of culture-positive bacterial infections in patients with cirrhosis worldwide, with significant geographical variation [6]. Multidrug resistance is increasing particularly among Gram-negative bacteria, with *Escherichia coli*, the most prevalent infectious agent in cirrhosis, being a leading contributor [6]. *E. coli* resistance is frequently driven by the acquisition of extended-spectrum beta-lactamases (ESBL) [7].

Infections caused by MDRO in cirrhosis are associated with poorer clinical outcomes, including lower rates of infection resolution, increased mortality and a higher incidence of septic shock and organ failure [6,8,9]. This organ failure often manifests as acute-on-chronic liver failure (ACLF), a severe condition that is unique to cirrhosis [10]. A delay in adequate antibiotic treatment seems to be the main reason for the poorer outcomes in patients with infections caused by MDRO [6,9,11]. However, it remains unclear whether other factors, such as a different inflammatory response against MDRO, could also contribute to the poor prognosis. This question is especially relevant in patients with cirrhosis. These patients are not only predisposed to infections because of their immune deficiencies. CAID also encompasses high-grade and low-grade systemic inflammation, together with an increased production of proinflammatory cytokines [2]. Among these cytokines, IL-6 and IL-8 are of particular relevance because their excessive production has been related to a worse prognosis and ACLF development in patients with cirrhosis [12,13].

Data regarding the in vitro inflammatory response against MDRO are scarce and contradictory. While some authors have reported an increased production of proinflammatory cytokines induced by multidrug-resistant (MDR) *E. coli* in comparison with susceptible strains [14], other studies have found a lower production [15]. It is relevant to elucidate whether the immune response elicited by MDRO is different from that against susceptible strains. This difference could have significant therapeutic implications in the future, not only in high-risk groups such as patients with cirrhosis but also in the general population.

We aimed to determine whether the in vitro immune response differs between MDR *E. coli* and susceptible *E. coli* strains isolated from infected patients with cirrhosis.

## 2. Materials and Methods

### 2.1. Bacterial Isolates and Growth Conditions

Twelve *E. coli* isolates from infected patients with cirrhosis were randomly selected and used for the experiments: six from ascitic fluid in the context of a spontaneous bacterial peritonitis (SBP) and six from blood in the context of bacteremia. SBP was defined as a bacterial infection of ascitic fluid without any intra-abdominal surgically treatable source of infection, while bacteremia refers to a bloodstream infection according to current guidelines [16].

Identification of *E. coli* isolates was initially suspected based on colony morphology and growth on chromogenic agar (CPS agar; bioMérieux, Marcy-l’Étoile, France). Species confirmation was subsequently performed using MALDI-TOF mass spectrometry (Bruker Daltonics GmbH & Co., KG, Bremen, Germany).

Antimicrobial susceptibility testing was performed using the disk diffusion method in accordance with the guidelines established by the European Committee on Antimicrobial Susceptibility Testing (EUCAST). Mueller–Hinton agar was used for all assays, and incubation was carried out at 35 ± 1 °C for 16–20 h in aerobic conditions. The results were interpreted following EUCAST breakpoints applicable to the years of the study (EUCAST v8.1, 2018; EUCAST v9.0, 2019; and EUCAST v10.0, 2020) [17,18,19].

Isolates showing resistance or reduced susceptibility to third-generation cephalosporins (e.g., cefotaxime, ceftazidime, ceftriaxone) were simultaneously screened for potential ESBL production by observing synergy between cephalosporin disks and an amoxicillin-clavulanic acid (20/10) disk. Ceftazidime (30 µg), cefotaxime (30 µg) and cefepime (30 µg) disks were placed 20–30 mm from the central clavulanate disk on Mueller–Hinton agar plates inoculated with bacterial suspensions adjusted to 0.5 McFarland turbidity. Enhancement of the inhibition zone between the clavulanate disk and any of the cephalosporin disks was interpreted as indicative of ESBL activity [20]. Quality control was performed throughout using *Klebsiella pneumoniae* ATCC 700603 (ESBL-positive) and *Escherichia coli* ATCC 25922 (ESBL-negative).

Six of the twelve *E. coli* isolates were ESBL producers and the other six were susceptible to antibiotics.

### 2.2. Bacterial Extracts Isolations

Bacterial strains were fractionated into surface protein and DNA.

#### 2.2.1. Surface Protein Isolation

Surface protein was isolated for each strain from 50 mL overnight liquid cultures (18 h) in duplicates, corresponding to the stationary growth phase. The method was adapted from previously published protocols, with slight modifications [21,22]. Cells were harvested by centrifugation (20 min, 10,000× *g*, 4 °C) and the resulting pellets were resuspended in 0.01 N NaOH solution. Protease inhibitors ethylenediaminetetraacetic acid (EDTA) and phenylmethylsulphonyl fluoride (PMSF) (Sigma-Aldrich, St. Louis, MO, USA) were added to all the solutions at final concentrations of 5 mM and 1 mM, respectively. Suspensions were gently agitated for 30 min at 37 °C in an orbital rotary agitator (Stuart SB3, Staffordshire, UK). After incubation and centrifugation, the supernatants containing the protein extracts were filtered (0.45 µm) and stored at −20 °C until further use. The protein concentration was measured using the QubitTM Protein Assay Kit (Thermo Fisher Scientific, Waltham, MA, USA) prior to their use.

#### 2.2.2. Bacterial DNA Isolation

DNA extraction was performed for each strain from 1.5 mL broth cultures using the GenElute™ Bacterial Genomic DNA Kit (Sigma-Aldrich, Dorset, UK) following the manufacturer’s instructions. The DNA concentration was assessed using the QubitTM DNA Assay Kit (Thermo Fisher Scientific, Waltham, MA, USA) and stored at −80 °C.

### 2.3. Peripheral Blood Mononuclear Cells (PBMC) Isolation and Stimulation with Bacterial Extracts

PBMC were isolated from 10 mL of peripheral blood obtained from healthy donors using a Ficoll-Histopaque gradient and cryopreserved in liquid nitrogen. Cell concentrations were adjusted to 2 × 10^6^ PBMC/mL in RPMI medium (Dominique Dutscher, Bernolsheim, France) supplemented with 5% fetal bovine serum (Biological Industries, Beit Haemek, Israel), 1% penicillin/streptomycin, and 1% glutamine (Biowest, Nuaillé, France). This medium is hereafter referred to as complete-RPMI.

In preliminary experiments, we stimulated PBMC with surface protein extracts at concentrations of 0.02, 0.2, and 2 µg/mL in complete-RPMI medium in a final volume of 200 µL in 96-well culture plates (Thermo Fisher Scientific, Waltham, MA, USA). For DNA extracts, we tested concentrations of 0.2 and 2 µg/mL. PBMC in complete-RPMI alone served as the negative control. PBMC stimulated with lipopolysaccharide (LPS-EB ultrapure 5 × 10^6^ EU—InvivoGen, San Diego, CA, USA) at 1 μg/mL in complete-RPMI medium was used as the positive control. PBMC were incubated for 2 and 5 days at 37 °C. For the final experiments we used surface protein extracts at 0.2 µg/mL and DNA extracts at 2 µg/mL because these concentrations performed best in preliminary tests. We incubated PBMC for two days because we aimed to assess the innate immune response rather than the adaptative response.

Because the yield of PBMC isolated from each healthy donor was insufficient to culture all 12 *E. coli* extracts we used extracts from *Escherichia coli* ATCC^®^ 25922, a non-pathogenic clinical isolate from the American Type Culture Collection to standardize the results for all cultures.

### 2.4. Determination of Cytokine Concentration and Ratio

Preliminary analysis of supernatants was conducted using a bead-based multiplex immunoassay ProcartaPlex™ Human Inflammation Panel, 20 plex (Thermo Fisher Scientific, Waltham, MA, USA). From among the 20 cytokines analyzed for the final experiments, we selected a battery of nine cytokines on the basis of their clinical relevance in patients with cirrhosis [2,12,13] and/or their role in innate immune response. This battery of cytokines captures key aspects of the immune response to microorganisms, encompassing pro-inflammatory, regulatory, and chemotactic functions, while limiting the number of analytes to ensure analytical robustness and comparability across samples. Specifically, we analyzed interferon-gamma (IFN-γ), IL-1β, IL-10, IL-12p70, monocyte chemoattractant protein-1 (MCP-1), IL-8/CXCL8, IL-6, macrophage inflammatory protein-1 (MIP-1α), and MIP-1β.

A ProcartaPlex™ Human MMP Panel, 5 plex (Thermo Fisher Scientific, Waltham, MA, USA) was used to measure interferon-gamma (IFN-γ), IL-1β, IL-10, IL-12p70, and monocyte chemoattractant protein-1 (MCP-1). We used specific ELISA kits for IL-8/CXCL8 (Mabtech, Nacka Strand, Sweden), IL-6 (ImmunoTools, Friesoythe, Germany), macrophage inflammatory protein (MIP)-1α and MIP-1β (PeproTech, Thermo Fisher Scientific, USA), because these cytokines were saturated in the ProcartaPlex™.

The lower limits of quantification were as follows: 11 pg/mL for IFN-γ, 2.76 pg/mL for IL-1β, 1.56 pg/mL for IL-10, 7.32 pg/mL for IL-12p70, 3.08 pg/mL for MCP-1, 4 pg/mL for IL-8, 6.1 pg/mL for IL-6 and 8 pg/mL for MIP-1α and MIP-1β. Supernatants were diluted 1/50 for IFN-γ, IL-1β, IL-10, IL-12p70 and MCP-1, 1/200 for IL-8 and IL-6, and 1/30 for MIP-1α and MIP-1β.

We normalized cytokine concentrations (in pg/mL) by calculating the ratio of cytokine production from pathogenic *E. coli* to that from standard *E. coli* ATCC^®^ 25922 using the formula:

$$ratio=\frac{Pathogenic\;E.\;coli\;cytokine\;concentration-negative\;control\;cytokine\;concentration\;}{E.\;coli\;{ATCC}^{®}\;25922\;cytokine\;concentration-negative\;control\;cytokine\;concentration}$$

We then compared the cytokine ratios between MDR and susceptible *E. coli*. We also compared the cytokine ratios between the different healthy donors and between the site of isolation of *E. coli* (ascitic fluid or blood).

### 2.5. Statistical Analyses

Statistical analyses were conducted using Graph Pad Prism 9 software (GraphPad, Boston, MA, USA) and 2023 IBM SPSS Statistics for Windows, version 29.0.1.1 (IBM Corp., Armonk, NY, USA). The Shapiro–Wilk test was applied to test the normality of data distribution. Results are reported as percentages, median (IQR), and mean ± standard deviation. To test comparisons between groups we used the Fisher’s exact test, Mann–Whitney test, Student’s *t*-test, multivariable linear regression analysis, and the Bonferroni post-hoc test. Significance was established at a two-sided *p*-value lower than 0.05.

## 3. Results

Surface-associated protein extract and DNA extracts were obtained from multidrug-resistant *E. coli* (n = 6) and antibiotic-susceptible *E. coli* (n = 6) strains isolated from patients with cirrhosis, and from a reference *E. coli* strain (ATCC^®^ 25922). *E. coli* extracts were used to stimulate in vitro PBMC from healthy donors (n = 8).

Table 1 shows the clinical characteristics of the patients from whom the *E. coli* were isolated. There were no statistically significant differences in clinical and analytical characteristics between the MDR *E. coli* group and the susceptible *E. coli* group. Both groups were predominantly men, with similar mean ages (69 ± 10 vs. 68 ± 13 years, respectively). The etiology of cirrhosis was mostly alcohol-related and metabolic dysfunction-associated steatotic liver disease (MASLD), with impaired liver function at the infection diagnosis: Child–Pugh scores of 9 and MELD 23 ± 5.1 vs. 20 ± 9.3, respectively. There was a non-significant trend to higher C-reactive protein levels and white blood cell count in patients infected by MDR *E. coli*. The MDR *E. coli* group had a non-significant higher proportion of previous antibiotic prophylaxis (66.7% vs. 33.3%). Bacteremia was more common in the MDR *E. coli* group, while SBP was more common in the susceptible *E. coli* group. Nosocomial infections were exclusively associated with MDR *E. coli*. Both groups had similar rates of acute kidney injury, cirrhosis decompensation, ACLF, and 30-day mortality.

**Figure 1:**
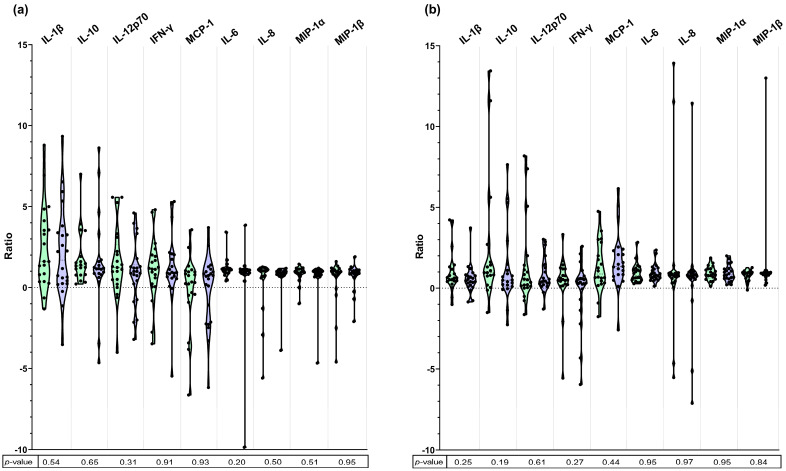
Violin plots showing standardized ratio of cytokine production in response to *E. coli* surface protein extract (**a**) and DNA extract (**b**), according to bacterial antibiotic susceptibility (antibiotic-susceptible in green, MDR in blue). Data are presented as individual results and median with IQR. Normality was assessed with the Shapiro–Wilk test; Student’s *t*-test or Mann–Whitney test were applied accordingly. No statistically significant differences between susceptible and MDR strains were detected in the production of any cytokine.

We compared the cytokine production between MDR *E. coli* and antibiotic-susceptible *E. coli* (Figure 1 and Table S1 and Table S2 in the Supplementary Materials). No statistically significant differences were detected in the production of any cytokine between MDR and susceptible strains.

Multivariable linear regression analysis confirmed this finding and revealed that the individual healthy donor significantly influenced the production of several cytokines (Table 2). Specifically, IL-1β, IL-10, IL-12p70, MCP-1, IL-8, MIP-1α, and MIP-1β levels in cultures stimulated with surface protein extracts showed statistically significant differences among healthy donors. Similarly, the individual healthy donor was associated with different production of IFN-γ, IL-1β, IL-10, IL-12p70, IL-8, IL-6, MIP-1α, and MIP-1β in cultures stimulated with DNA extracts. No significant differences were observed among healthy donors for IFN-γ and IL-6 levels in response to surface extracts, nor for MCP-1 levels in response to DNA extracts.

**Figure 2:**
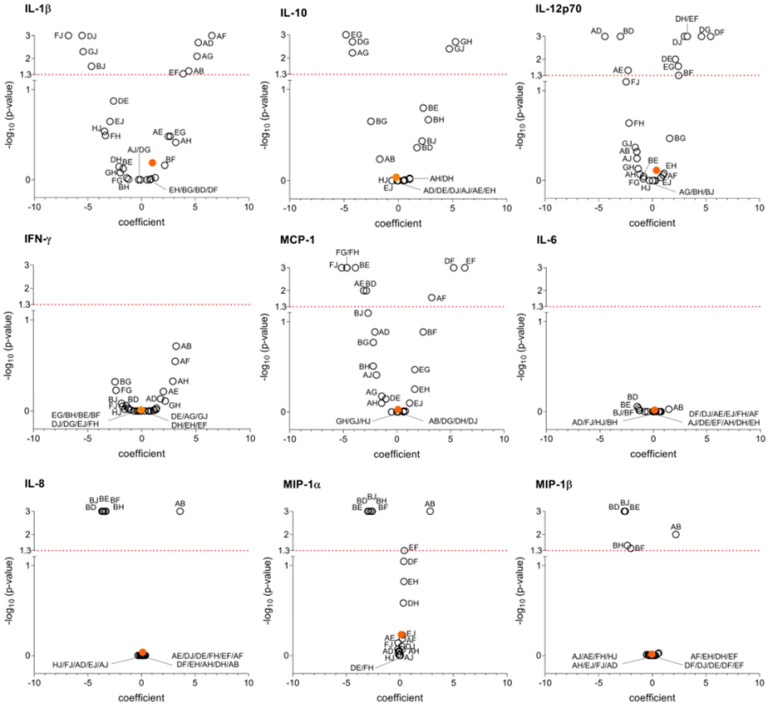
Volcano plots showing cytokine production in response to *E. coli* surface protein extract. Coefficients indicate the magnitude of the difference between antibiotic-susceptible and MDR *E. coli* (indicated by an orange dot), and between each healthy donor and every other healthy donor individually (represented by a white circle, with each donor labeled by a capital letter). *p*-values using multivariable lineal regression with Bonferroni correction for each coefficient are represented as −log_10_. A value of −log_10_ higher than 1.3 corresponds to a *p*-value < 0.05 (red dot line) and indicates statistical significance.

**Figure 3:**
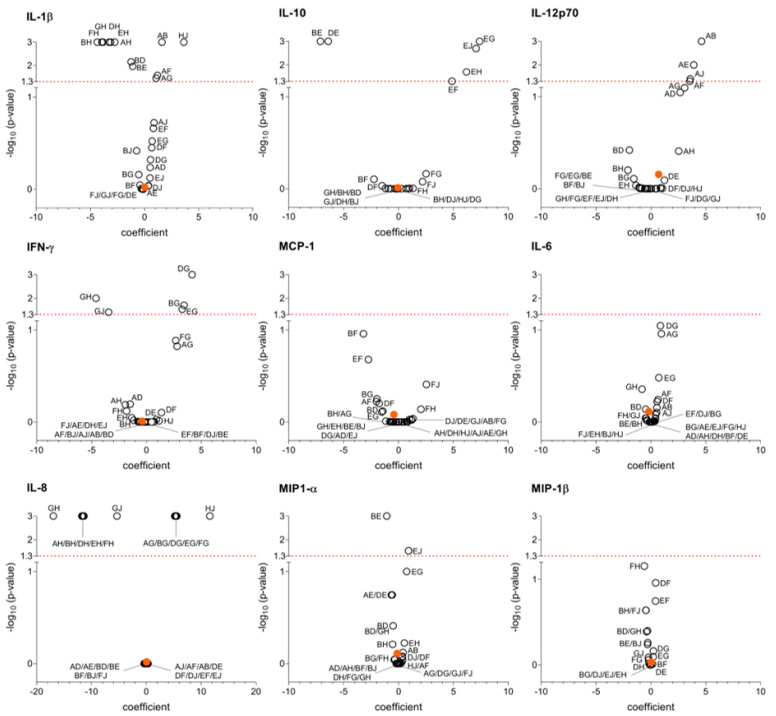
Volcano plots showing cytokine production in response to *E. coli* DNA extract. Coefficients indicate the magnitude of the difference between antibiotic-susceptible and MDR *E. coli* (indicated by an orange dot), and between each healthy donor and every other healthy donor individually (represented by a white circle, with each donor labeled by a capital letter). *p*-values using multivariable lineal regression with Bonferroni correction for each coefficient are represented as −log_10_. A value of −log_10_ higher than 1.3 corresponds to a *p*-value < 0.05 (red dot line) and indicates statistical significance.

To further explore these findings, we generated volcano plots (Figure 2 and Figure 3) to illustrate the coefficients and *p*-values with Bonferroni correction for cytokine production between susceptible and MDR strains and the variability among healthy donors. For example, in Figure 2, regarding IL-1β, donor J exhibited a different cytokine production pattern to that of the other donors. For IL-10, donor G showed the greatest difference. In contrast, no significant differences were observed among donors for IFN-γ.

Among the cytokines analyzed, the production levels were highest for IL-1β in surface protein extracts (ratio of 1.70 (0.24–3.49) for MDR strains vs. 1.61 (0.48–3.98) for susceptible strains, *p* = 0.54) and for IL-10 and MCP-1 in DNA extracts (ratios of 0.50 (0.13–1.39) vs. 0.96 (0.47–2.16), *p* = 0.19, and 1.34 (0.78–2.37) vs. 0.88 (0.36–2.63), *p* = 0.44, respectively) (Figure 1 and Tables S1 and S2 in the Supplementary Materials).

**Figure 4:**
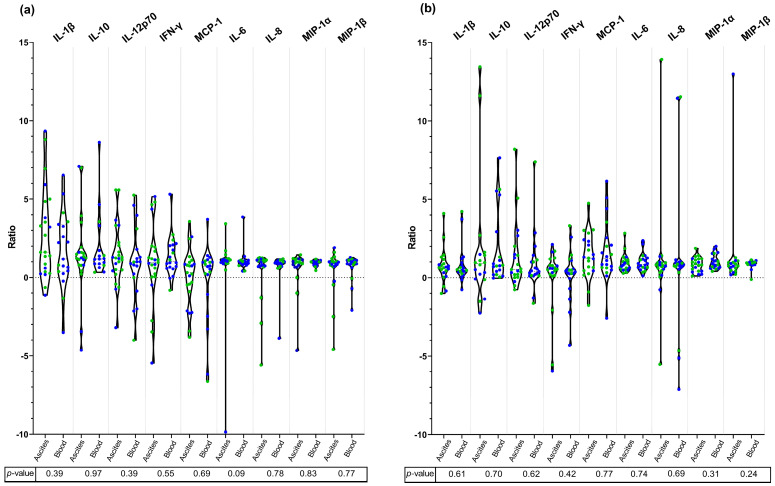
Violin plots showing the standardized ratio of cytokine production in response to *E. coli* surface protein extract (**a**) and DNA extract (**b**), according to the site of infection (ascites vs. blood). Antibiotic-susceptible strains are represented by green dots and MDR strains by blue dots. Data are presented as individual results and median with IQR. Normality was assessed with the Shapiro–Wilk test, Student’s *t*-test, or Mann–Whitney test were applied accordingly. No statistically significant differences were detected between ascites and blood in the production of any cytokine.

Lastly, we studied whether the type of infection, and consequently the site of bacterial isolation, influenced the immune response. We observed no significant differences in cytokine production based on the source of infection (ascites vs. blood) (Figure 4 and Tables S3 and S4 in the Supplementary Materials).

## 4. Discussion

The main finding in the present study was that the in vitro cytokine production by PBMC from healthy donors challenged with *E. coli* isolated from patients with cirrhosis was similar in MDR and susceptible strains. In addition, the production of most cytokines differed among individual healthy donors.

The rising prevalence of infections caused by MDRO worldwide is a serious threat to global public health, especially in patients with cirrhosis [6]. The link between MDR infections and higher mortality observed in previous reports [23,24] could have several non-exclusive explanations, such as delays in appropriate antibiotic treatment [6,9,11], intrinsic host factors [25], and bacterial virulence factors [26]. However, a different elicited immune response between MDR and susceptible bacteria could also contribute to the worse prognosis in the former. Few studies have evaluated in vitro how immunity responds to MDR *E. coli* through cytokine production. Bristianou et al. studied the early release of cytokines by human monocytes from healthy donors stimulated by *E. coli* obtained from the urine of women with acute pyelonephritis [14]. They observed a higher production of TNF, IL-6, and IL-8 by MDR *E. coli* than by susceptible strains, while the opposite occurred for IL-12. In contrast, Auger et al. found that beta-lactam-resistant *E. coli* strains isolated from urine or blood incubated with whole blood from healthy donors induced significantly lower levels of TNF-α and IL-1β than susceptible strains [15]. This discrepancy with Bristianou et al.’s results was attributed to the use of whole blood instead of monocytes.

In our study the battery of cytokines was selected on the basis of their clinical relevance in patients with cirrhosis [2,12,13] and/or their role in innate immune response to microorganisms, the target of our experimental approach. This battery included IFN-γ, IL-1β, IL-10, IL-12p70, MCP-1, MIP-1α, MIP-1β, IL-6, and IL-8 and allowed us to analyze essential components of the immune response, covering pro-inflammatory, regulatory, and chemotactic functions. We did not find statistically significant differences in the production of any of these cytokines between MDR *E. coli* and susceptible strains. It is of note that the production of IL-6 and IL-8, pro-inflammatory cytokines with a relevant prognostic value in cirrhosis [12,13], did not differ between the two groups of *E. coli* strains. These results suggest that the worse prognosis of MDR *E. coli* infections is more likely attributable to factors such as delayed appropriate antibiotic treatment [6,9] or a patient’s poorer baseline status than to a different immune response elicited by the bacteria. Moreover, our data in vitro do not support the use of specific immune modulation as a therapeutic strategy for sepsis caused by MDR *E. coli*. However, we cannot exclude the potential role of therapeutic immune modulation in vivo or in other contexts [27].

The mechanisms of antibiotic resistance in *E. coli* are mainly located in the bacterial envelope. These mechanisms include antibiotic inactivating/modifying enzymes (such as carbapenemases and ESBLs), outer membrane porin remodeling, enhanced efflux pump action to actively transport antibiotics out of the cell, and alteration of antibiotic target sites (e.g., lipid A region of lipopolysaccharide) [28]. In the present study, all the MDR *E. coli* were ESBL-producing strains. Therefore, it seemed interesting to study the differences between MDR and susceptible *E. coli* in the immune response elicited specifically by the surface bacterial extract. However, although the surface components of bacteria play a key role in triggering the human immune response, other bacterial components such as CpG DNA also stimulate this response. In effect, circulating bacterial DNA is associated with the proinflammatory state [29] and a worse prognosis [30] in patients with cirrhosis. Notably, bacterial surface and DNA components stimulate the immune system through different pathways. Surface lipopolysaccharide binds to Toll-like receptor (TLR) 4 and lipoproteins to TLR 2/TLR 1 whereas bacterial CpG-containing DNA binds to TLR 9 [31,32,33,34]. The immune response to surface components could therefore differ from that elicited by DNA extracts. For this reason, in contrast to the studies by Bristianou and Auger who used the whole bacteria [14,15], we analyzed the surface extract and the DNA extract separately, and we did not find relevant differences in the immune response between MDR *E. coli* and susceptible *E. coli* in any. However, whole bacteria may simultaneously activate multiple pattern recognition receptors, resulting in a synergistic cytokine regulation [35]. This effect could lead to an immune response different from that elicited by surface and DNA extracts separately. Future studies comparing whole bacteria with their isolated components could provide further insight into these interactions.

In this study, we used PBMC from healthy donors rather than from patients with cirrhosis to minimize variability and potential confounding factors. Patients with cirrhosis exhibit high heterogeneity in baseline immune alterations, history of infections, liver failure severity, comorbidities and pharmacological treatments. All these factors could mask potential differences in cytokine production between MDR and susceptible *E. coli*, especially given the limited number of strains analyzed. Therefore, we decided to use PBMC from healthy donors, a theoretically more homogeneous population. Nevertheless, among the healthy donors, we observed high variability in the production of most cytokines, highlighting the role of genetic polymorphisms and environmental factors in modulating inflammatory pathways. For instance, a genome-wide association study revealed innate immunity was strongly influenced by several genetic loci, while factors such as smoking, age, and sex were shown to predominantly influence adaptive immunity [36]. Previous microbial exposure may also influence systemic immune responses, including cytokine production. It is important to consider that patients with cirrhosis exhibit a different immune response compared to healthy subjects. In fact, an exaggerated in vitro response to bacterial products has been reported in patients with cirrhosis compared to healthy controls, particularly in the production of interferons, IL-6 and IL-8 [37]. Therefore, it remains unknow whether the similar cytokine profile found between MDR and susceptible *E. coli* strains in PBMC from healthy controls can be extrapolable to PBMC from patients with cirrhosis.

The cytokines with the highest production were IL-1β in surface extracts and IL-10 and MCP-1 in DNA extracts. IL-1β is a proinflammatory cytokine that has an important role in the immune response after the initial host recognizes bacterial infection and enhances phagocyte recruitment and function [38]. MCP-1 is a chemokine that plays a key role in recruiting monocytes and polymorphonuclear cells [39]. In contrast, IL-10 has been reported to act as an anti-inflammatory cytokine that protects the host from a harmful uncontrolled immune response [38]. Nevertheless, IL-10 can also exhibit immunostimulatory properties under certain conditions [40].

The site from which *E. coli* was isolated (ascitic fluid vs. blood) did not significantly influence the in vitro immune response. This observation aligns with the understanding that SBP and spontaneous bacteremia—the most characteristic infections in cirrhosis—share the common pathophysiological pathway of bacterial translocation, with bacteria originating from the intestinal lumen moving to various extraintestinal sites [41].

Our study has certain limitations. First, the number of bacteria is limited. However, our intention was to mimic an ex vivo clinical scenario using strains isolated from infected patients with cirrhosis and to evaluate a comprehensive battery of cytokines. Second, we used multiple healthy donors instead of relying on a single donor for all experiments. The variability generated between these different donors could therefore limit the comparison between MDR and susceptible strains. However, we tried to control this potential bias by multivariable linear regression analysis. This approach of using several healthy donors increased the generalizability of our results and allowed us to observe the role of donor variability in the immune response. Third, the PBMC were obtained from healthy donors rather than patients with cirrhosis. Nonetheless, this serves as a preliminary approach to understanding how the general population responds to MDR *E. coli* and provides a foundation for future studies with PBMC from patients with cirrhosis. It would also be valuable to extend this approach to other MDR bacteria. Fourth, we used different cytokine quantification techniques to evaluate the results because some cytokines were saturated in the multiplex immunoassay. While this introduced variability, it allowed us to assess a broader range of cytokine responses. Lastly, our study is based on in vitro experiments. While valuable to understand certain aspects of immune responses under well-defined conditions, such experiments cannot fully replicate the complexity and dynamic nature of the immune environment in vivo.

## 5. Conclusions

Within the limitations of the sample size, we conclude that there were no significant differences in in vitro cytokine production between MDR and antibiotic-susceptible *E. coli* strains isolated from infected patients with cirrhosis. This result suggests that the poor prognosis associated with MDR infections is likely driven by factors other than differences in immune response. Furthermore, our data in vitro do not support the use of specific immune modulation as a targeted therapeutic approach for MDR infections. Notably, we observed that immune response was strongly influenced by interindividual variability among healthy donors. This result underscores the importance of considering host variability in shaping immune response to infections. Future research should explore the underlying mechanisms of this variability and its clinical implications, particularly in vulnerable populations such as patients with cirrhosis.

## Figures and Tables

**Table 1:** Clinical and analytical characteristics of patients with cirrhosis infected by MDR *E. coli* and antibiotic-susceptible *E. coli* from whom bacterial isolates were obtained. Results are expressed as percentages or mean ± standard deviation. Normality was assessed with the Shapiro–Wilk test; Student’s *t*-test or Mann–Whitney test were applied accordingly. Fisher’s exact test was used for comparisons of categorical variables. MASLD: metabolic dysfunction-associated steatotic liver disease; MetALD: metabolic and alcohol-related liver disease; MELD: model for end-stage liver disease; SBP: spontaneous bacterial peritonitis; HE: hepatic encephalopathy; ACLF: acute-on-chronic liver failure.

	Infection by MDR *E. coli* n = 6	Infection by Susceptible *E. coli* n = 6	*p*-Value
Sex, M/F, n (%)	6 (100%)/0	4 (66.7%)/2 (33.3%)	0.45
Age, (years)	69 ± 10	68 ± 13	0.48
Etiology of cirrhosis, n (%)			0.46
Alcohol-related liver disease	2 (33.3%)	1 (16.7%)	
MASLD	3 (50%)	2 (33.3%)	
MetALD	1 (16.7%)	1 (16.7%)	
Alcohol + Autoimmune	0	2 (33.3%)	
Child–Pugh score at infection diagnosis, (points)	9 ± 2.3	9 ± 1.7	0.50
MELD score at infection diagnosis, (points)	23 ± 5.1	20 ± 9.3	0.23
C-reactive protein (mg/dL)	103.0 ± 63.4	93.7 ± 50.0	0.78
White blood cell count (×109/L)	10.9 ± 5.5	8.2 ± 4.4	0.38
Antibiotic prophylaxis, n (%)	4 (66.7%)	2 (33.3%)	0.57
Type of infection, n (%)			0.56
Bacteremia	4 (66.7%)	2 (33.3%)	
SBP	2 (33.3%)	4 (66.7%)	
Site of acquisition, n (%)			0.07
Community-acquired	2 (33.3%)	3 (50%)	
Healthcare associated	2 (33.3%)	3 (50%)	
Nosocomial	2 (33.3%)	0	
Acute kidney injury at infection diagnosis, n (%)	2 (33.3%)	3 (50%)	1
Decompensation of cirrhosis, n (%)	6 (100%), of which:	5 (83.3%), of which:	1
Ascites	3 (50%)	5 (100%)	
Variceal bleeding	1 (16.7%)	0	
Ascites + HE	2 (33.3%)	0	
ACLF, n (%)	1 (16.7%)	2 (33.3%)	1
30-day mortality, n (%)	1 (16.7%)	0	1

**Table 2:** Multivariable linear regression analysis for each cytokine. Coefficients indicate the magnitude of the difference between antibiotic-susceptible vs. MDR *E. coli*; results are expressed as β coefficient and 95% confidence intervals. *p*-values for antibiotic-susceptible vs. MDR *E. coli* and among healthy donors are reported.

		Surface Protein Extract		DNA Extract	
		Antibiotic-Susceptible vs. MDR *E. coli*	Healthy Donors	Antibiotic-Susceptible vs. MDR *E. coli*	Healthy Donors
IL-1β	coefficient (95% CI)	1.01 (−3.51–5.53)	-	0.03 (−1.15–1.21)	-
	*p*-value	0.65	<0.001 *	0.96	<0.001 *
IL-10	coefficient (95% CI)	−0.19 (−3.84–3.47)	-	−0.14 (−5.78–5.48)	-
	*p*-value	0.92	0.01 *	0.96	0.002 *
IL-12p70	coefficient (95% CI)	0.39 (−2.28–3.05)	-	0.71 (−3.07–4.49)	-
	*p*-value	0.77	<0.001 *	0.70	0.01 *
IFN-γ	coefficient (95% CI)	−0.07 (−4.61–4.47)	-	−0.04 (−3.6–3.53)	-
	*p*-value	0.97	0.18	0.98	0.008 *
MCP-1	coefficient (95% CI)	0.11 (−2.95–3.18)	-	−0,42 (−4.32–3.48)	-
	*p*-value	0.94	<0.001 *	0,83	0.40
IL-6	coefficient (95% CI)	0.11 (−4.81–5.02)	-	−0.18 (−1.40–1.05)	-
	*p*-value	0.96	0.93	0.77	0.045 *
IL-8	coefficient (95% CI)	0.11 (−2.08–2.31)	-	0.06 (−2.36–2.50)	-
	*p*-value	0.92	<0.001 *	0.96	<0.001 *
MIP-1α	coefficient (95% CI)	0.13 (−0.36–0.62)	-	−0.13 (−1.10–0.84)	-
	*p*-value	0.58	<0.001 *	0.78	0.01 *
MIP-1β	coefficient (95% CI)	−0.04 (−2.19–2.11)	-	0.03 (−0.59–0.64)	-
	*p*-value	0.97	0.004 *	0.93	0.01 *

(*): statistically significant *p*-values.

## Data Availability

The original contributions presented in this study are included in the article/Supplementary Material. Further inquiries can be directed to the corresponding author.

## Supplementary Materials

The following supporting information can be downloaded at: https://www.mdpi.com/article/10.3390/microorganisms13051164/s1, Table S1: Standardized ratio of cytokine production in response to *E. coli* surface protein extract (a) and DNA extract (b), according to bacterial antibiotic susceptibility. Results are expressed as median (IQR). Normality was assessed with the Shapiro–Wilk test; Student’s *t*-test or Mann–Whitney test were applied accordingly; Table S2: Absolute concentrations (pg/mL) of cytokine production in response to *E. coli* surface protein extract (a) and DNA extract (b), according to bacterial antibiotic susceptibility. Results are expressed as median (IQR). Normality was assessed with the Shapiro–Wilk test; Student’s *t*-test or Mann–Whitney test were applied accordingly; Table S3: Standardized ratio of cytokine production in response to *E. coli* surface protein extract (a) and DNA extract (b), according to the site of infection (ascites vs. blood). Results are expressed as median (IQR). Normality was assessed with the Shapiro–Wilk test; Student’s *t*-test or Mann–Whitney test were applied accordingly; Table S4: Absolute concentrations (pg/mL) of cytokine production in response to *E. coli* surface protein extract (a) and DNA extract (b), according to the site of infection (ascites vs. blood). Results are expressed as median (IQR). Normality was assessed with the Shapiro–Wilk test; Student’s *t*-test or Mann–Whitney test were applied accordingly.

## Abbreviations

The following abbreviations are used in this manuscript:

ACLF	Acute-on-chronic liver failure;
CAID	Cirrhosis-associated immune dysfunction;
HE	Hepatic encephalopathy;
ESBL	Extended-spectrum—betalactamases;
EUCAST	European Committee on Antimicrobial Susceptibility Testing;
IFN-γ	Interferon-gamma;
MASLD	Metabolic dysfunction-associated steatotic liver disease;
MCP-1	Monocyte chemoattractant protein-1;
MDR	Multidrug-resistant;
MDRO	Multidrug-resistant organisms;
MetALD	Metabolic and alcohol-related liver disease;
MIP	Macrophage inflammatory protein;
PBMC	Peripheral blood mononuclear cells;
SBP	Spontaneous bacterial peritonitis;
TLR	Toll-like receptor.
