# Supplementary material for: In Vitro Immune Response of Mononuclear Cells to Multidrug-Resistant Escherichia coli
Source: Microorganisms. 2025 May 20;13(5):1164. doi: 10.3390/microorganisms13051164 (PMC12114291; doi:10.3390/microorganisms13051164)
Supplement: Supplementary file 1 [file microorganisms-13-01164-s001.zip › microorganisms-3615121-supplementary.pdf]

SUPPLEMENTARY MATERIAL

In Vitro Immune Response of Mononuclear Cells to Multidrug-Resistant *Escherichia coli*

Berta Cuyàs [1,2,3], Elisabet Cantó [4], Elisabet Sanchez-Ardid [1,3], Elisenda Miró [5,6], Edilmar Alvarado-Tapias [1,2,3], Eva Román [1,2,3], Maria Poca [1,2,3], Ferran Navarro [5,6], Andreu Ferrero-Gregori [4], Maria Àngels Escorsell [1,2,3], Silvia Vidal [4], German Soriano [1,2,3*]

Table S1. Standardized ratio of cytokine production in response to *E. coli* surface protein extract (a) and DNA extract (b), according to bacterial antibiotic susceptibility. Results are expressed as median (IQR). Normality was assessed with the Shapiro–Wilk test; Student's t-test or Mann–Whitney test were applied accordingly. *MDR*: multidrug-resistant.

a) Surface protein extract

	IL-1β	IL-10	IL-12p70	IFN-γ	MCP-1	IL-6	IL-8	MIP-1α	MIP-1β
**Antibiotic-susceptible *E. coli***	1.61 (0.48-3.98)	1.33 (0.77-1.92)	1.24 (0.34-2.67)	1.14 (0.14-1.97)	0.34 (-0.45-1.00)	1.09 (0.90-1.19)	1.03 (0.67-1.12)	0.98 (0.79-1.15)	0.96 (0.68-1.10)
**MDR *E. coli***	1.70 (0.24-3.49)	1.16 (0.81-1.69)	0.94 (-0.02-1.42)	0.92 (0.57-2.04)	0.72 (-2.16-1.02)	0.96 (0.87-1.10)	0.90 (0.74-1.05)	0.94 (0.77-1.06)	0.87 (0.71-1.08)
**p-value**	0.54	0.65	0.31	0.91	0.93	0.20	0.50	0.51	0.95

b) DNA extract

	IL-1β	IL-10	IL-12p70	IFN-γ	MCP-1	IL-6	IL-8	MIP-1α	MIP-1β
**Antibiotic-susceptible *E. coli***	0.63 (0.42-1.28)	0.96 (0.47-2.16)	0.45 (0.06-1.35)	0.52 (0.23-1.19)	0.88 (0.36-2.63)	0.78 (0.50-1.21)	0.78 (0.36-0.95)	0.86 (0.59-1.21)	0.88 (0.54-0.98)
**MDR *E. coli***	0.46 (0.13-0.79)	0.50 (0.13-1.39)	0.44 (0.24-1.45)	-0.37 (-0.37-0.68)	1.34 (0.78-2.37)	0.82 (0.62-1.22)	0.79 (0.55-0.99)	0.82 (0.59-1.20)	0.90 (0.76-0.97)
**p-value**	0.25	0.19	0.61	0.27	0.44	0.95	0.97	0.95	0.84

Table S2. Absolute concentrations (pg/mL) of cytokine production in response to *E. coli* surface protein extract (a) and DNA extract (b), according to bacterial antibiotic susceptibility. Results are expressed as median (IQR). Normality was assessed with the Shapiro–Wilk test; Student's t-test or Mann–Whitney test were applied accordingly. *MDR*: multidrug-resistant.

a) Surface protein extract

	IL-1β	IL-10	IL-12p70	IFN-γ	MCP-1	IL-6	IL-8	MIP-1α	MIP-1β
**Antibiotic-susceptible *E. coli***	1756.00 (921.84-3306.32)	372.75 (219.19-520.82)	7.72 (5.99-9.59)	93.89 (45.74-384.82)	1049.16 (868.65-1200.25)	10942.86 (7609.52-14323.81)	108278.90 (78342.86-148628.60)	13950.00 (12200.00-16550.00)	6866.25 (5778.75-8553.75)
**MDR *E. coli***	1549.85 (862.32-2881.91)	385.07 (175.90-519.66)	7.14 (5.93-8.82)	114.13 (51.26-243.72)	964.98 (857.88-1160.71)	14228.57 (8323.81-18323.81)	133485.70 (88914.29-148914.30)	14000.00 (12825.00-15925.00)	7278.75 (5253.75-11066.25)
**p-value**	0.65	0.98	0.58	1	0.83	0.21	0.28	0.80	0.34

b) DNA extract

	IL-1β	IL-10	IL-12p70	IFN-γ	MCP-1	IL-6	IL-8	MIP-1α	MIP-1β
**Antibiotic-susceptible *E. coli***	1147.02 (892.79-1504.24)	361.26 (94.46-489.94)	7.38 (6.24-8.35)	53.97 (29.50-174.92)	913.74 (819.33-1158.62)	7085.71 (3966.67-13014.29)	100485.72 (71778.41-148200.03)	12200.00 (8300.00-13636.58)	6898.75 (5628.75-7785.00)
**MDR *E. coli***	1267.37 (929.55-1927.82)	253.99 (134.02-403.92)	7.38 (6.56-8.39)	64.73 (33.50-160.46)	938.16 (812.72-1170.84)	13085.71 (5990.48-21514.29)	147843.80 (90771.43-184342.85)	14200.00 (10600.00-16650.00)	7635.00 (6082.16-10241.25)
**p-value**	0.55	0.98	0.99	0.93	0.55	0.07	0.09	0.14	0.08

Table S3. Standardized ratio of cytokine production in response to *E. coli* surface protein extract (a) and DNA extract (b), according to the site of infection (ascites vs. blood). Results are expressed as median (IQR). Normality was assessed with the Shapiro–Wilk test; Student's t-test or Mann–Whitney test were applied accordingly.

a) Surface protein extract

	**IL-1β**	**IL-10**	**IL-12p70**	**IFN-γ**	**MCP-1**	**IL-6**	**IL-8**	**MIP-1α**	**MIP-1β**
**Ascites**	1.61 (0.36–4.85)	1.24 (0.78–1.68)	1.22 (0.44–2.18)	0.96 (0.05–1.93)	0.31 (–0.91–0.97)	1.09 (0.91–1.18)	1.03 (0.71–1.11)	0.95 (0.69–1.14)	0.95 (0.70–1.02)
**Blood**	1.17 (0.37–3.39)	1.17 (0.82–2.51)	0.98 (0.00–1.79)	1.03 (0.60–2.07)	0.72 (–0.77–1.03)	0.94 (0.79–1.09)	0.92 (0.79–1.08)	0.97 (0.79–1.04)	0.96 (0.72–1.09)
**p-value**	0.39	0.97	0.39	0.55	0.69	0.09	0.78	0.83	0.77

b) DNA extract

	**IL-1β**	**IL-10**	**IL-12p70**	**IFN-γ**	**MCP-1**	**IL-6**	**IL-8**	**MIP-1α**	**MIP-1β**
**Ascites**	0.64 (0.20–1.03)	0.89 (–0.09–1.52)	0.53 (0.12–1.75)	0.58 (0.18–0.99)	1.25 (0.44–2.37)	0.82 (0.50–1.18)	0.76 (0.38–0.96)	0.82 (0.41–1.21)	0.75 (0.45–1.00)
**Blood**	0.46 (0.28–0.91)	0.76 (0.19–2.93)	0.41 (0.13–1.10)	0.39 (0.03–0.96)	1.22 (0.63–2.43)	0.85 (0.61–1.24)	0.81 (0.57–0.98)	0.86 (0.64–1.45)	0.93 (0.84–0.98)
**p-value**	0.61	0.70	0.62	0.42	0.77	0.74	0.69	0.31	0.24

Table S4. Absolute concentrations (pg/mL) of cytokine production in response to *E. coli* surface protein extract (a) and DNA extract (b), according to the site of infection (ascites vs. blood). Results are expressed as median (IQR). Normality was assessed with the Shapiro–Wilk test; Student's t-test or Mann–Whitney test were applied accordingly.

a) Surface protein extract

	**IL-1β**	**IL-10**	**IL-12p70**	**IFN-γ**	**MCP-1**	**IL-6**	**IL-8**	**MIP-1α**	**MIP-1β**
**Ascites**	1195.07 (905.54-1539.05)	307.60 (96.05-496.04)	7.38 (6.72-8.31)	74.19 (29.10-200.45)	897.57 (809.94-1103.92)	6419.05 (3847.62-14276.20)	103200.00 (68057.14-149093.35)	11300.00 (6600.00-15100.00)	6472.50 (5628.75-7822.50)
**Blood**	1489.94 (912.36-2939.02)	322.11 (202.34-562.47)	7.38 (6.27-8.73)	102.27 (36.58-234.94)	922.07 (795.77-1074.56)	14228.57 (8085.71-18466.67)	124057.10 (86914.29-152628.60)	14300.00 (12325.00-16175.00)	7128.75 (5516.25-10110.00)
**p-value**	0.87	0.96	0.83	0.54	0.178	0.65	0.53	0.50	0.42

b) DNA extract

	**IL-1β**	**IL-10**	**IL-12p70**	**IFN-γ**	**MCP-1**	**IL-6**	**IL-8**	**MIP-1α**	**MIP-1β**
**Ascites**	1861.07 (792.28-3032.74)	413.20 (207.01-476.47)	7.38 (5.80-9.07)	93.89 (56.72-322.58)	1077.53 (920.49-1400.00)	11276.19 (7966.67-14728.57)	109771.40 (82914.29-147485.75)	13850.00 (12575.00-16550.00)	7241.25 (5778.75-8478.75)
**Blood**	1261.04 (904.79-2022.48)	240.11 (175.98-403.98)	7.05 (6.16-8.39)	47.70 (35.57-145.10)	961.39 (831.28-1193.16)	11514.28 (6728.57-17823.81)	148628.55 (91146.17-177128.60)	13550.00 (12225.00-15800.00)	7766.25 (6603.75-10728.75)
**p-value**	0.38	0.86	0.49	0.59	0.31	0.15	0.23	0.06	0.05
